# Nobiletin alleviates cisplatin-induced ototoxicity via activating autophagy and inhibiting NRF2/GPX4-mediated ferroptosis

**DOI:** 10.1038/s41598-024-55614-4

**Published:** 2024-04-03

**Authors:** Wenao Song, Li Zhang, Xiaolin Cui, Rongrong Wang, Jingyu Ma, Yue Xu, Yan Jin, Dawei Wang, Zhiming Lu

**Affiliations:** 1 Department of Clinical Laboratory, Shandong Provincial Hospital, Shandong University, Jinan, 250021 China; 2 Department of Clinical Laboratory, Shandong Provincial Hospital Affiliated to Shandong First Medical University, Jinan, 250021 China; 3 Department of Orthopedic, Shandong Provincial Hospital Affiliated to Shandong First Medical University, Jinan, 250021 China; 4 Department of Otolaryngology-Head and Neck Surgery, Shandong Provincial Hospital, Shandong University, Jinan, 250021 China

**Keywords:** Drug discovery, Medical research

## Abstract

Nobiletin, a citrus polymethoxy flavonoid with antiapoptotic and antioxidative properties, could safeguard against cisplatin-induced nephrotoxicity and neurotoxicity. Cisplatin, as the pioneer of anti-cancer drug, the severe ototoxicity limits its clinical applications, while the effect of nobiletin on cisplatin-induced ototoxicity has not been identified. The current study investigated the alleviating effect of nobiletin on cisplatin-induced ototoxicity and the underlying mechanisms. Apoptosis and ROS formation were evaluated using the CCK-8 assay, Western blotting, and immunofluorescence, indicating that nobiletin attenuated cisplatin-induced apoptosis and oxidative stress. LC3B and SQSTM1/p62 were determined by Western blotting, qPCR, and immunofluorescence, indicating that nobiletin significantly activated autophagy. Nobiletin promoted the nuclear translocation of NRF2 and the transcription of its target genes, including *Hmox1*, *Nqo1*, and ferroptosis markers (*Gpx4*, *Slc7a11*, *Fth*, and *Ftl*), thereby inhibiting ferroptosis. Furthermore, RNA sequencing analysis verified that autophagy, ferroptosis, and the NRF2 signaling pathway served as crucial points for the protection of nobiletin against ototoxicity caused by cisplatin. Collectively, these results indicated, for the first time, that nobiletin alleviated cisplatin-elicited ototoxicity through suppressing apoptosis and oxidative stress, which were attributed to the activation of autophagy and the inhibition of NRF2/GPX4-mediated ferroptosis. Our study suggested that nobiletin could be a prospective agent for preventing cisplatin-induced hearing loss.

Hearing loss is widely considered the most prevalent sensory deficits, which is mainly attributed to aging, noise trauma, genetic mutations, and ototoxic drugs^1^. Among these factors, cisplatin serves as a representative agent, which is extensively applied to investigate the mechanisms underlying the exogenous ototoxicity. Cisplatin, as the pioneer of anti-cancer drug, has been widely applied to treat various solid neoplasms. Unfortunately, the cisplatin chemotherapy causes adverse side effects, including ototoxicity. Due to the bilateral, symmetrical, irreversible, cumulative, and dose-dependent characteristics of cisplatin-induced sensorineural deafness, ototoxicity has attracted increasing attention^2^. The histopathological changes of cisplatin ototoxicity are characterized by the apoptosis of mechanosensory hair cells, the damage of spiral ganglion neurons (SGNs), and the atrophy of stria vascularis^3^. The reactive oxygen species (ROS) overproduction is commonly considered the mechanism of cisplatin ototoxicity^4,5^. Subsequently, the ROS accumulation leads to diverse types of cell death, including apoptosis, necroptosis, ferroptosis, and autophagy^6–9^.

Autophagy plays crucial roles in preserving cellular integrity through clearing dysfunctional organelles, degrading protein aggregates, and recycling the breakdown products^10^. Increasing evidence has shown that autophagy is implicated in the development and modulation of cisplatin ototoxicity. Rapamycin, as an autophagy activator, prevented hair cell apoptosis accompanied by the enhanced expressions of microtubule-associated protein 1 light chain 3-II (LC3-II) and Beclin-1^11^. Conversely, 3-methyladenine (3-MA), as an autophagy inhibitor, aggravated the severity of cisplatin-induced auditory cell injury^12^. Both LC3 and sequestosome 1 (SQSTM1/p62) have been extensively utilized as autophagic activity biomarkers. SQSTM1/p62 functions as a selective substrate and cargo receptor for autophagic degradation and promotes the transcription of nuclear factor erythroid 2-related factor 2 (NRF2)^13^.

NRF2 acts as a redox-sensitive nuclear factor to defend cells against oxidative stresses and preserve dynamic equilibrium primarily through modulating the transcriptional activity of antioxidant response element (ARE)^14,15^. Numerous studies have indicated that pretreatment with NRF2 stimulators, e.g., flunarizine^16^, ebselen^17^, ginkgolide B^18^, and phloretin^19^, protected hair cells from cisplatin by decreasing oxidative stresses, restraining proapoptotic transcription, and promoting the activation of cytoprotective enzymes, particularly, the antioxidant enzymes. Furthermore, NRF2 not only maintains cellular redox homeostasis, but also functions as a key regulator of ferroptosis^20^. Moreover, ferroptosis is involved in the initiation and development of cisplatin-induced auditory cell impairment^9^. Recently, a study has clarified that the activation of NRF2 inhibits ferroptosis, thereby significantly mitigating oxaliplatin-induced ototoxicity^21^.

Nobiletin, derived from citrus fruit peels, is a polymethoxy flavonoid with multiple pharmaceutical properties, in particular, the antioxidative and antiapoptotic effects. Studies have shown that through enhancing autophagy, nobiletin was revealed to ameliorate the oxidative stress triggered by Alzheimer's disease and hepatic ischemia and reperfusion^22,23^. Nobiletin was previously reported to mitigate sepsis-associated acute liver injury by inhibiting the NRF2/glutathione peroxidase 4 (GPX4)-mediated ferroptosis^24^. Furthermore, previous studies demonstrated that nobiletin could ameliorate nephrotoxicity and neurotoxicity caused by cisplatin^25,26^.

To date, no research has reported the effect of nobiletin on cisplatin-induced hearing loss. Therefore, the goal of current study was to determine the potential protective effect of nobiletin on cisplatin-elicited ototoxicity and the underlying molecular mechanisms.

## Results

### Nobiletin protects HEI-OC1 cells and cochlear explants against cisplatin-induced cytotoxicity

**Figure 1:**
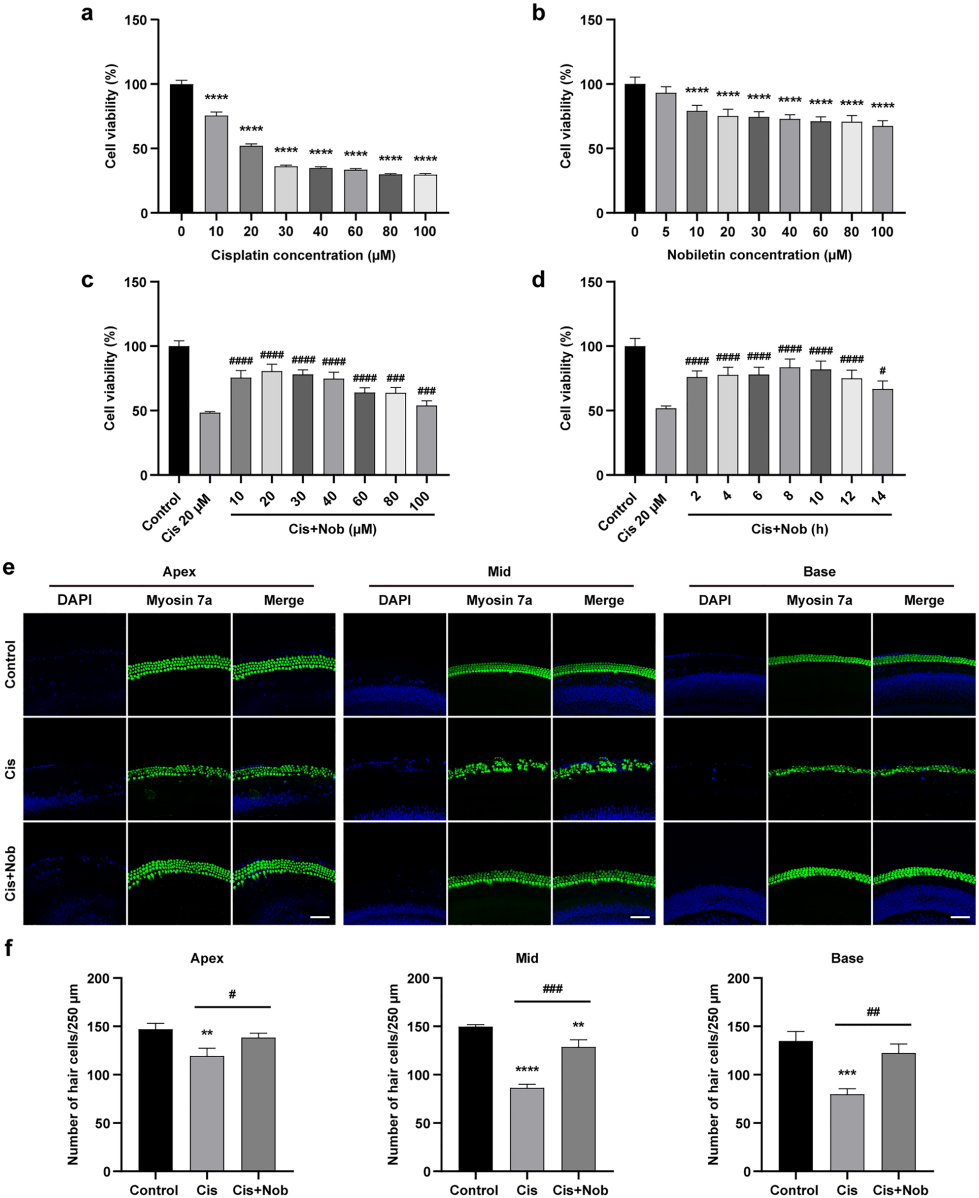
Nobiletin protects HEI-OC1 cells and cochlear explants against cisplatin-induced cytotoxicity. (**a**) Cell viability of HEI-OC1 cells treated with the designated doses of cisplatin for 24 h based on CCK-8 assay (n = 5). (**b**) Cell viability of HEI-OC1 cells treated with the designated doses of nobiletin for 24 h based on CCK-8 assay (n = 5). (**c**) Cell viability of HEI-OC1 cells after the pretreatment with incremental doses of nobiletin for 3 h and co-treated with 20 µM cisplatin for 24 h based on CCK-8 assay (n = 4). (**d**) Cell viability of HEI-OC1 cells after the pretreatment with 20 µM nobiletin for incremental times and co-treated with 20 µM cisplatin for 24 h based on CCK-8 assay (n = 4). (**e**) Representative images of Myosin 7a and DAPI immunofluorescence staining of hair cells in different cochlear regions under various treatments. (**f**) Quantification of hair cells in different cochlear regions (n = 3). Scale bars = 50 µm. *P < 0.05, **P < 0.01, ***P < 0.001, and ****P < 0.0001 vs. control group; # P < 0.05, ## P < 0.01, and ### P < 0.001 vs. cisplatin group.

The results of cell counting kit-8 (CCK-8) assay showed that the cell viability declined in a dose-dependent manner after the House Ear Institute-Organ of Corti 1 (HEI-OC1) cells were subjected to treatment of escalating doses of cisplatin for 24 h. Under the treatment of 20 µM cisplatin for 24 h, the cell viability was decreased by approximately 50% (Fig. 1a). Therefore, cisplatin at 20 µM was used in the subsequent in vitro experiments. To evaluate the potential cytotoxicity of nobiletin, the HEI-OC1 cells were exposed to incremental doses of nobiletin for 24 h. The results indicated that nobiletin reduced the cell viability at doses above 10 µM (Fig. 1b). To ascertain the protection of nobiletin against cytotoxicity caused by cisplatin (20 µM for 24 h), the HEI-OC1 cells were pretreated with incremental doses of nobiletin for 3 h. The optimal protective effect was attained with nobiletin at 20 µM compared with the treatment of cisplatin alone (Fig. 1c). To confirm the optimum pretreatment time of nobiletin in mitigating cytotoxicity caused by cisplatin (20 µM for 24 h), the HEI-OC1 cells were pretreated with 20 µM nobiletin for varied amounts of time. The results of CCK-8 assay showed that the optimum protective effect was achieved at 8 h of pretreatment with 20 µM nobiletin (Fig. 1d). Therefore, treatment of nobiletin at 20 µM for 8 h was applied in the following experiments.

To verify that nobiletin could make the identical impact on hair cells from P4 C57BL/6 mice cochleae, the cochlear explants were incubated and treated in the following groups: the control group (no treatment), the cisplatin group (20 µM cisplatin stimulus for 24 h), and the cisplatin-plus-nobiletin group (20 µM nobiletin pretreatment for 8 h, followed by 20 µM cisplatin stimulus for 24 h). The results of immunofluorescence staining indicated that the hair cells were orderly arranged with cellular integrity retained in the control group. Conversely, there was a significant loss of cochlear hair cells after the administration of cisplatin, manifesting as a disordered arrangement and aberrant morphology, particularly in the middle and basal regions of cochlea. Nevertheless, the cochlear hair cells substantially rose following nobiletin pretreatment (Fig. 1e, f). These results suggested that nobiletin protected against cytotoxicity caused by cisplatin.

### Nobiletin suppresses cisplatin-induced apoptosis in HEI-OC1 cells and cochlear explants

**Figure 2:**
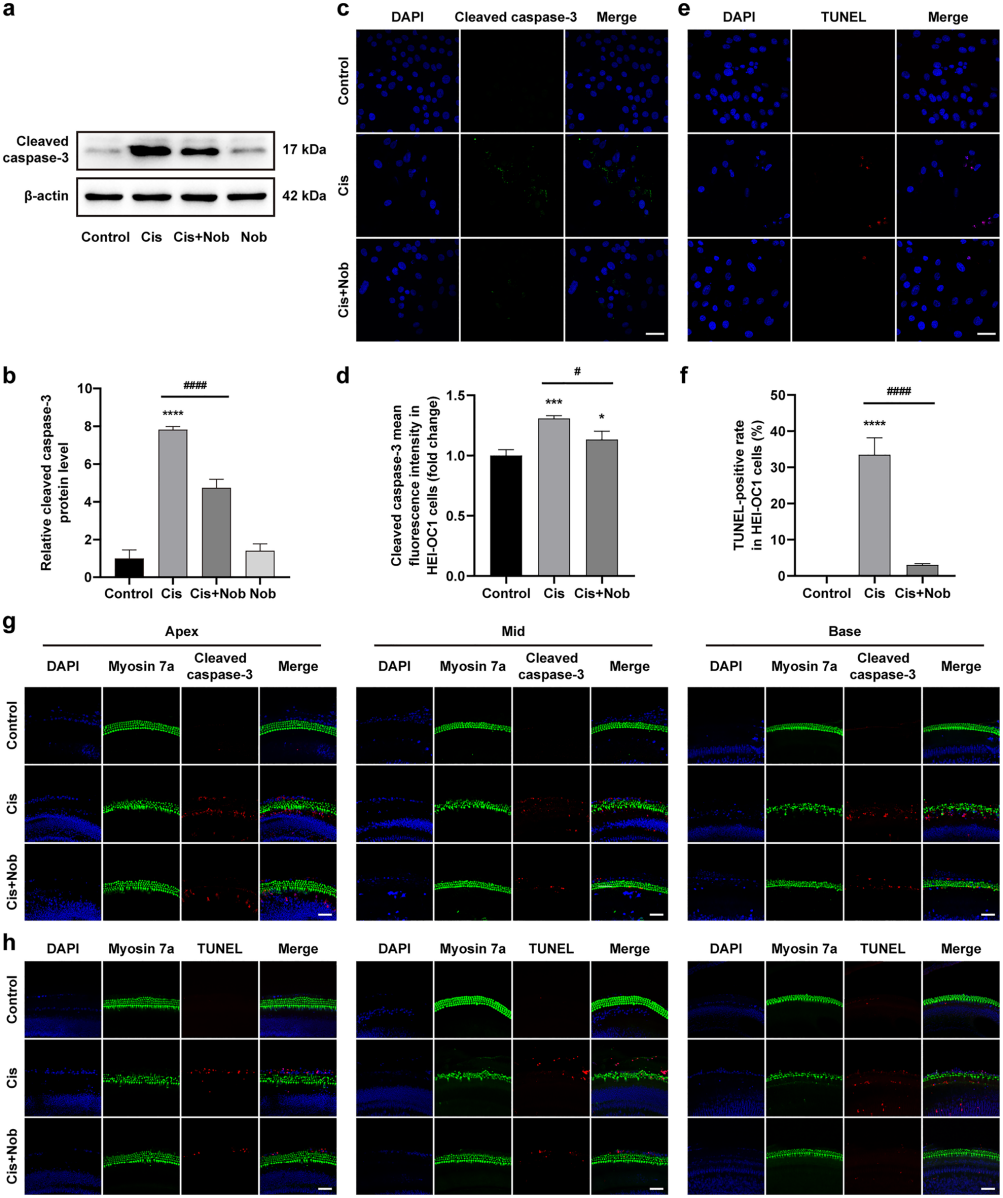
Nobiletin suppresses cisplatin-induced apoptosis in HEI-OC1 cells and cochlear explants. (**a, b**) Protein levels of cleaved caspase-3 in HEI-OC1 cells assessed by Western blotting (n = 3). The original blots are presented in Supplementary Fig. S1. (**c**) Representative images of cleaved caspase-3 immunofluorescence staining of HEI-OC1 cells under various treatments. (**d**) Quantitation of intensity levels of cleaved caspase-3 immunofluorescence shown in (**c**) (n = 3). (**e**) Representative images of TUNEL staining of HEI-OC1 cells under various treatments. (**f**) Quantification of TUNEL-positive cells shown in (**e**) (n = 3). (**g, h**) Representative images of cleaved caspase-3 immunofluorescence staining and TUNEL staining of hair cells in different cochlear regions under various treatments. Scale bar = 50 µm. *P < 0.05, **P < 0.01, ***P < 0.001, and **** P < 0.0001 vs. control group; # *P* < 0.05, ## *P* < 0.01, ### *P* < 0.001, and #### *P* < 0.0001 vs. cisplatin group.

The results of Western blotting confirmed that nobiletin was revealed with a significant suppressive effect on the cisplatin-induced increase in the level of cleaved caspase-3 in HEI-OC1 cells, and there was no significant difference in the levels of cleaved caspase-3 between the nobiletin alone group and the control group (Fig. 2a, b). The results of cleaved caspase-3 and TUNEL staining of HEI-OC1 cells manifested that cisplatin treatment caused a remarkable increase in the fluorescence intensity of cleaved caspase-3 and the number of TUNEL-positive cells. In contrast, the intensity of cleaved caspase-3 and TUNEL signals was notably weakened after the pretreatment of nobiletin (Fig. 2c–f). Similarly, the results of cleaved caspase-3 and TUNEL staining of cochlear explants from different regions indicated that the hair cells pretreated with nobiletin exhibited higher integrality, along with the lower fluorescence intensity of cleaved caspase-3 and fewer TUNEL-positive cells, compared to the treatment of cisplatin alone (Fig. 2g, h). These results suggested that nobiletin suppressed apoptosis caused by cisplatin.

### Nobiletin relieves cisplatin-induced oxidative damage in HEI-OC1 cells and cochlear explants

**Figure 3:**
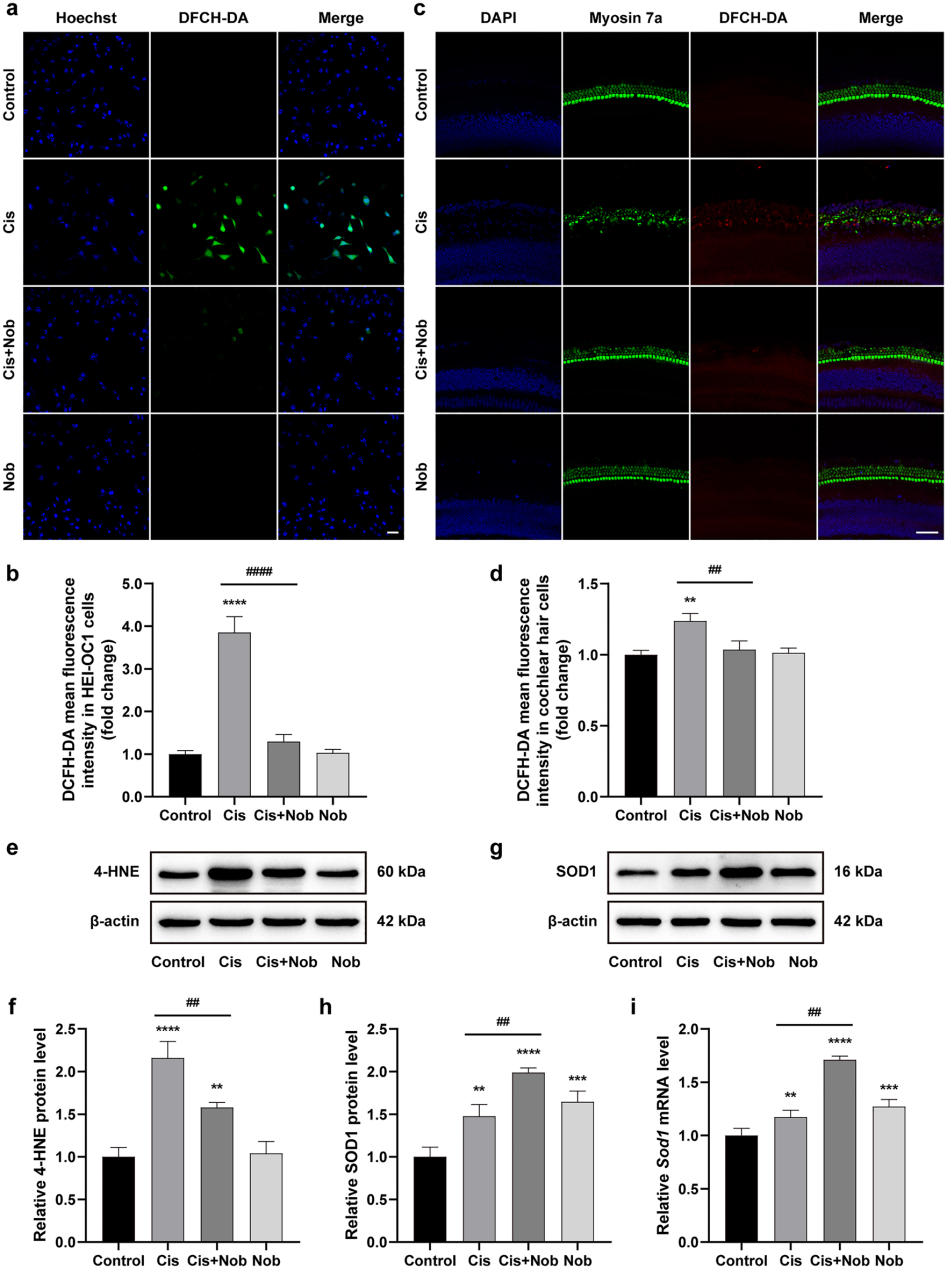
Nobiletin relieves cisplatin-induced oxidative damage in HEI-OC1 cells and cochlear explants. (**a**) Representative images of DCFH-DA and Hoechst 33,258 immunofluorescence staining of HEI-OC1 cells under various treatments. (**b**) Quantitation of intensity levels of DCFH-DA immunofluorescence shown in (**a**) (n = 3). (**c**) Representative images of DCFH-DA and Myosin 7a immunofluorescence staining of cochlear middle regions under various treatments. (**d**) Quantitation of intensity levels of DCFH-DA immunofluorescence shown in (**c**) (n = 3). (**e–h**) Protein levels of 4-HNE and SOD1 in HEI-OC1 cells based on Western blotting (n = 3). The original blots are presented in Supplementary Fig. S1. (**i**) The mRNA levels of gene encoding *Sod1* in HEI-OC1 cells based on qPCR (n = 3). Scale bar = 50 µm. *P < 0.05, **P < 0.01, ***P < 0.001, and ****P < 0.0001 vs. control group; #*P* < 0.05, ##*P* < 0.01, ###*P* < 0.001, and ####*P* < 0.0001 vs. cisplatin group.

The results showed that the florescence of DCFH-DA staining was considerably enhanced after cisplatin administration. Conversely, the florescence was substantially mitigated after nobiletin preconditioning in both HEI-OC1 cells and cochlear explants. Furthermore, treatment with nobiletin alone caused no significant change in fluorescence intensity (Fig. 3a–d). As the end product of lipid peroxidation in response to oxidative stress, 4-hydroxynonenal (4-HNE) is regarded as an indicator of free radical generation. The results of Western blotting demonstrated that the pretreatment of nobiletin drastically decreased the cisplatin-induced elevation of 4-HNE in HEI-OC1 cells. Additionally, the exclusive administration of nobiletin induced no significant alterations in the expression of 4-HNE (Fig. 3e,f). The results also revealed the compensatory up-regulation of superoxide dismutase 1 (SOD1), an antioxidant marker, in response to the exposure to cisplatin. The treatment of nobiletin alone resulted in a moderate elevation in the expression of SOD1. In contrast, pretreatment with nobiletin prior to cisplatin exposure significantly enhanced the expression of SOD1. These findings were further supported by the qPCR assay (Fig. 3g–i). These results suggested that nobiletin relieved cisplatin-induced oxidative stress.

### Nobiletin alleviates cisplatin-induced damage via activating autophagy

**Figure 4:**
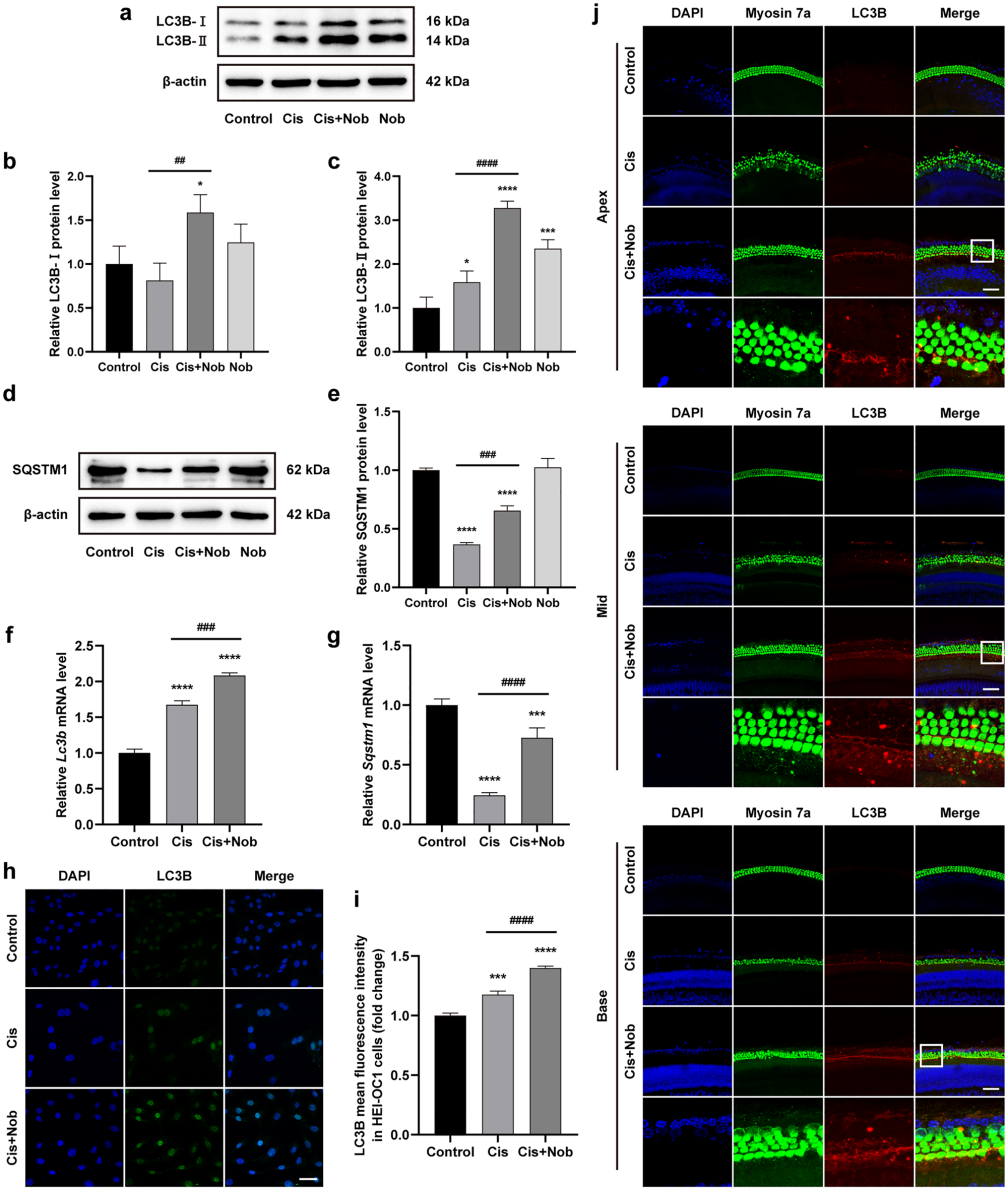
Nobiletin alleviates cisplatin-induced damage via activating autophagy. (**a–e**) Protein levels of LC3B and SQSTM1 in HEI-OC1 cells based on Western blotting (n = 3). The original blots are presented in Supplementary Fig. S1. (**f, g**) The mRNA levels of genes encoding *Lc3b* and *Sqstm1* in HEI-OC1 cells based on qPCR (n = 3). (**h**) Representative images of LC3B immunofluorescence staining of HEI-OC1 cells under various treatments. (**i**) Quantitation of intensity levels of LC3B immunofluorescence shown in (**h**) (n = 3). (**j**) Representative images of LC3B and Myosin 7a immunofluorescence staining of hair cells in different cochlear regions under various treatments. Scale bar = 50 µm. *P < 0.05, **P < 0.01, ***P < 0.001, and ****P < 0.0001 vs. control group; #*P* < 0.05, ##*P* < 0.01, ###*P* < 0.001, and ####*P* < 0.0001 vs. cisplatin group.

The results of Western blot demonstrated a slight elevation in the expression of LC3B-II and a reduction in the expression of SQSTM1/p62 following cisplatin damage, whereas treatment with nobiletin alone led to a moderate increase in the expression of LC3B-II, with no significant difference observed in the expression of SQSTM1/p62 compared to the control group. Notably, the pretreatment of nobiletin before cisplatin exposure markedly enhanced the levels of LC3B-II and SQSTM1/p62 in HEI-OC1 cells (Fig. 4a–e). The elevated level of SQSTM1/p62 could be ascribed to the hyperactivation of antioxidative gene encoding *Nrf2*, which mediated the expression of SQSTM1/p62 by creating a positive feedback loop^27^. These findings were further corroborated by the qPCR analysis (Fig. 4f,g). The results of immunofluorescence staining of HEI-OC1 cells revealed that the fluorescence of LC3B was substantially increased in nobiletin-pretreated cells, accompanied by evident punctate aggregation (Fig. 4h,i). Similarly, after the treatment of cisplatin, visible spotted autophagosomes were formed in cochlear explants, along with extensive degeneration and evident loss of hair cells. After the pretreatment of nobiletin, a large amount of spotted autophagosomes as well as more intense and condensed LC3B signals and more intact hair cells were observed at different areas of the cochlear explants (Fig. 4j). These results suggested that nobiletin alleviated cisplatin-induced damage via activating autophagy.

### Nobiletin activates NRF2-mediated pathway in HEI-OC1 cells and cochlear explants

**Figure 5:**
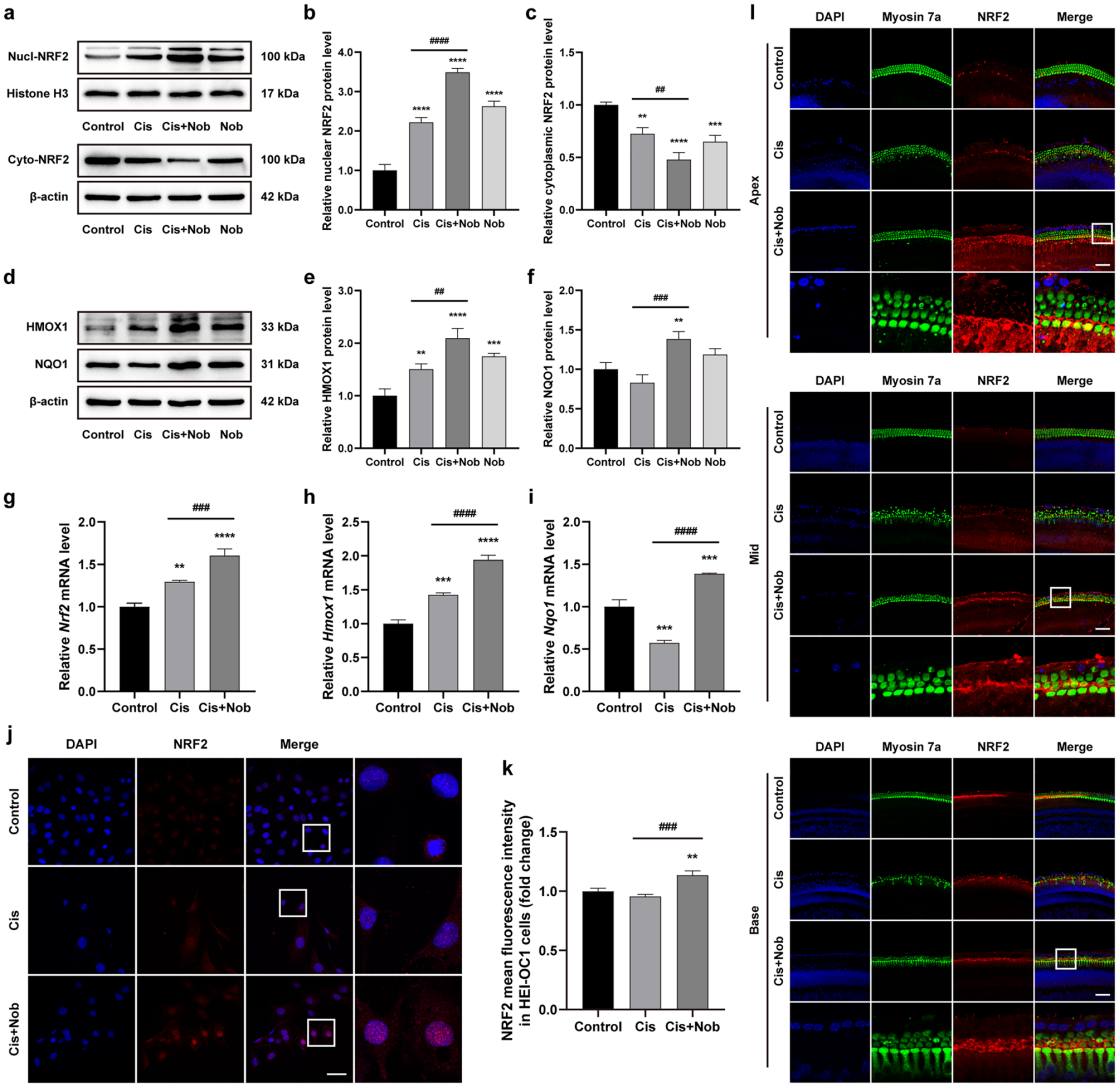
Nobiletin activates NRF2-mediated pathway in HEI-OC1 cells and cochlear explants. (**a–f**) Protein levels of nuclear NRF2 and cytoplasmic NRF2, HMOX1, and NQO1 in HEI-OC1 cells based on Western blotting (n = 3). The original blots are presented in Supplementary Fig. S2. (**g–i**) The mRNA levels of genes encoding *Nrf2*, *Hmox1*, and *Nqo1* in HEI-OC1 cells based on qPCR (n = 3). (**j**) Representative images of NRF2 immunofluorescence staining of HEI-OC1 cells under various treatments. (**k**) Quantitation of intensity levels of NRF2 immunofluorescence shown in (**j**) (n = 3). (**l**) Representative images of NRF2 and Myosin 7a immunofluorescence staining of hair cells in different cochlear regions under various treatments. Scale bar = 50 µm. *P < 0.05, **P < 0.01, ***P < 0.001, and ****P < 0.0001 vs. control group; #*P* < 0.05, ##*P* < 0.01, ###*P* < 0.001, and ####*P* < 0.0001 vs. cisplatin group.

The results of Western blotting suggested that, in comparison to the control group, there was a slight increase in the nuclear fraction of NRF2 and a concurrently mild decrease in the cytoplasmic fraction of HEI-OC1 cells after cisplatin exposure, accompanied by a subtle compensatory up-regulation of heme oxygenase 1 (HMOX1). However, pretreatment with nobiletin markedly facilitated nuclear translocation of NRF2 and enhanced the expressions of its target genes encoding *Hmox1* and NAD(P)H quinone oxidoreductase 1 (*Nqo1*) in the presence or absence of cisplatin, as particularly observed in the cisplatin-plus-nobiletin group (Fig. 5a–f). These results were further substantiated by the qPCR assay (Fig. 5g–i). The results of immunofluorescence in HEI-OC1 cells revealed a faint NRF2 labeling in the control group. After the treatment of cisplatin, the red fluorescence signal of NRF2 was slightly enhanced, whereas in nobiletin-pretreated cells, the red fluorescence intensity of NRF2 was substantially fortified, with evident nuclear accumulation of NRF2 observed (Fig. 5j,k). Similarly, the co-treatment of both nobiletin and cisplatin drastically promoted the nuclear translocation of NRF2 in cochlear explants (Fig. 5l). The findings suggested that nobiletin could activate NRF2-mediated pathway in both HEI-OC1 cells and cochlear hair cells.

### Nobiletin ameliorates cisplatin-induced injury via inhibiting ferroptosis

**Figure 6:**
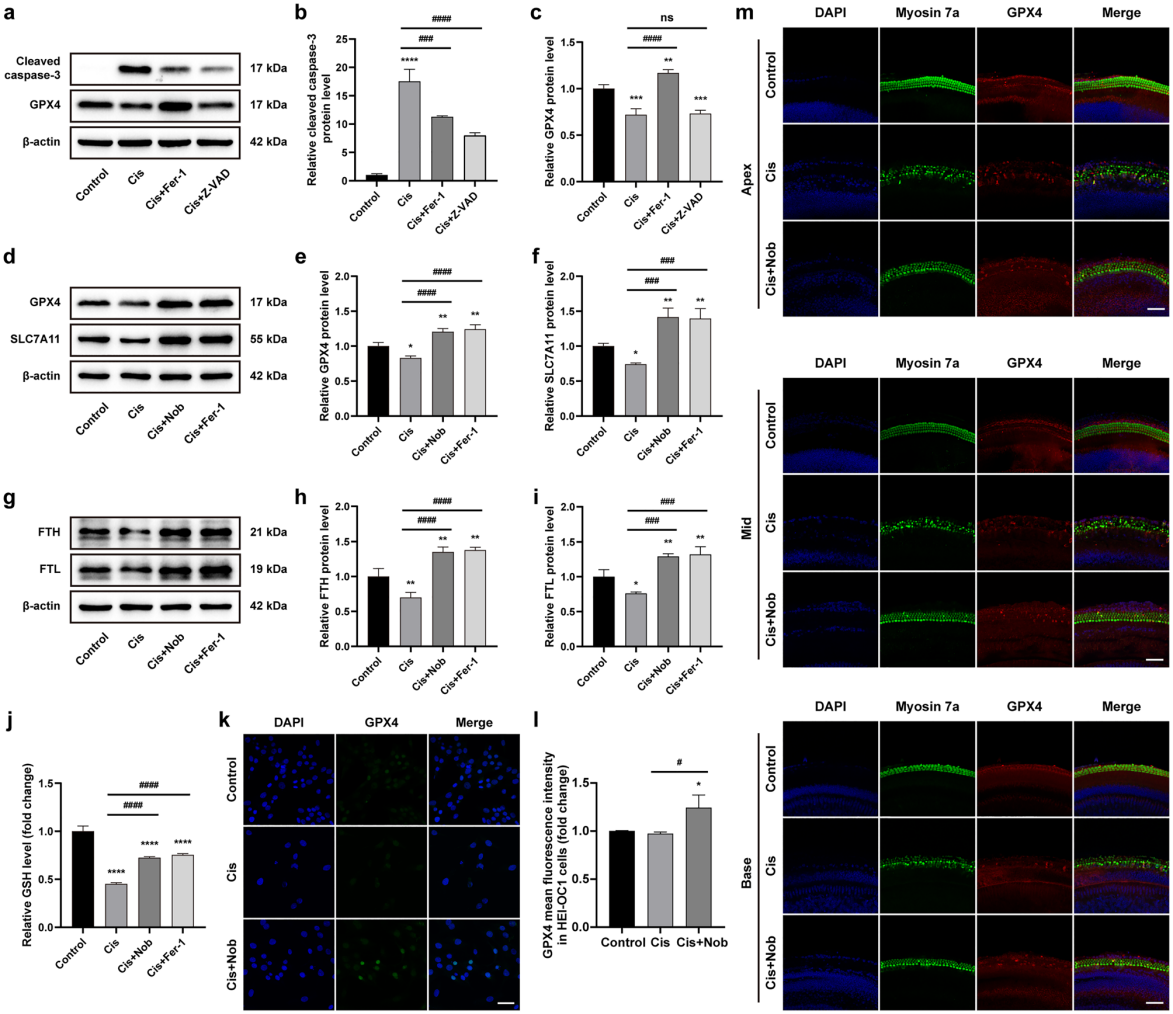
Nobiletin ameliorates cisplatin-induced injury via inhibiting ferroptosis. (**a–c**) Protein levels of cleaved caspase-3 and GPX4 in HEI-OC1 cells treated with cisplatin after pretreatment with Fer-1 or Z-VAD-FMK based on Western blotting (n = 3). The original blots are presented in Supplementary Fig. S3. (**d–i**) Protein levels of GPX4, SLC7A11, FTH, and FTL in HEI-OC1 cells treated with cisplatin after pretreatment with nobiletin or ferrostatin-1 based on Western blotting (n = 3). The original blots are presented in Supplementary Fig. S4. (**j**) Levels of GSH measured in HEI-OC1 cells under various treatments (n = 3). (**k**) Representative images of GPX4 immunofluorescence staining of HEI-OC1 cells under various treatments. (**l**) Quantitation of intensity levels of GPX4 immunofluorescence shown in (**k**) (n = 3). (**m**) Representative images of GPX4 and Myosin 7a immunofluorescence staining of hair cells in different cochlear regions under various treatments. Scale bar = 50 µm. *P < 0.05, **P < 0.01, ***P < 0.001, and ****P < 0.0001 vs. control group; #*P* < 0.05, ##*P* < 0.01, ###*P* < 0.001, and ####*P* < 0.0001 vs. cisplatin group.

To investigate the relationship between ferroptosis and apoptosis induced by cisplatin, ferrostatin-1 (Fer-1) and Z-VAD-FMK were selected as inhibitors to block ferroptosis and apoptosis, respectively. The results of Western blotting based on HEI-OC1 cells demonstrated that the expression of cleaved caspase-3 was elevated following cisplatin stimulus and markedly reduced after pretreatment with Fer-1 or Z-VAD-FMK. The cisplatin-induced decrease in the expression of GPX4 could be reversed by pretreatment with Fer-1, but not by Z-VAD-FMK (Fig. 6a–c). These results suggested that cisplatin-induced ferroptosis may occur prior to apoptosis, and the inhibition of ferroptosis could mitigate apoptosis.

We further explored the impact of nobiletin on cisplatin-induced ferroptosis compared with Fer-1. The results of Western blot and GSH assay demonstrated that the treatment of cisplatin led to a simultaneous reduction in the levels of ferroptosis-related biomarkers in HEI-OC1 cells, including GPX4, solute carrier family 7 member 11 (SLC7A11), ferritin heavy chain (FTH), ferritin light chain (FTL), and glutathione (GSH). However, pretreatment with nobiletin significantly reversed their declines, showing comparable effects as that of Fer-1 (Fig. 6d–j). The immunofluorescence staining was conducted to verify that the cisplatin stimulus weakened the fluorescence intensity of GPX4, while the pretreatment of nobiletin remarkably enhanced the level of GPX4 and effectively alleviated cellular damage in both HEI-OC1 cells and cochlear explants (Fig. 6k–m). These findings indicated that nobiletin ameliorated cisplatin-induced injury via inhibiting ferroptosis.

### Validation of targets and signaling pathways associated with the protection of nobiletin against cisplatin-induced ototoxicity using RNA sequencing

**Figure 7:**
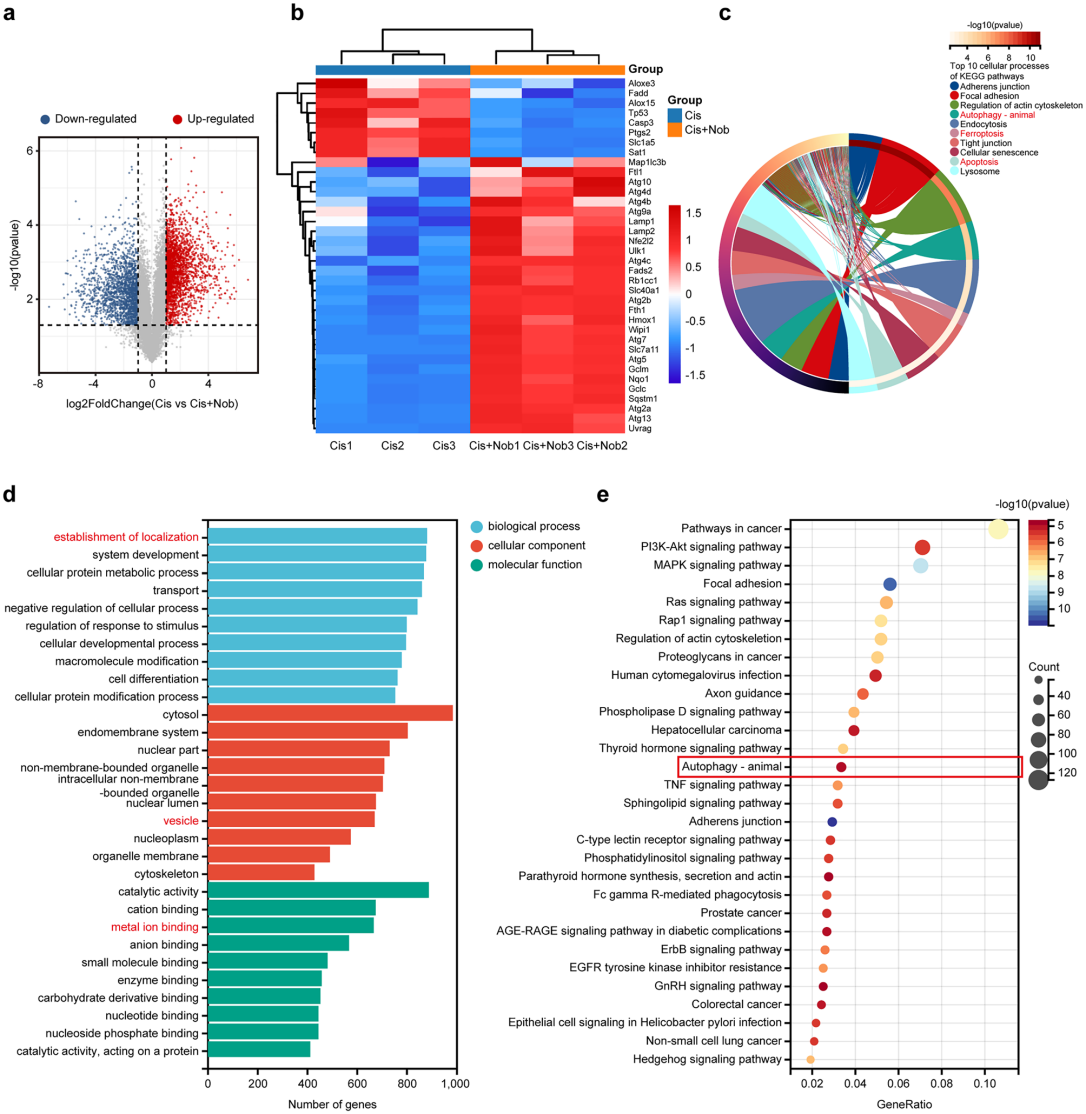
Validation of targets and signaling pathways associated with the protection of nobiletin against cisplatin-induced ototoxicity using RNA sequencing. (**a**) Volcanic map of differentially expressed genes (DEGs). (**b**) Heatmap of DEGs related to autophagy, ferroptosis, and NRF2 signaling pathway. (**c**) The top 10 cellular processes based on KEGG enrichment analysis. (**d**) GO annotation analysis based on the DEGs. (**e**) The top 30 pathways based on KEGG enrichment analysis.

Among the approximately 13,765 genes identified by RNA sequencing analysis, a total of 4,250 differentially expressed genes (DEGs) (2,437 up-regulated and 1,813 down-regulated) were revealed between cisplatin and cisplatin-plus-nobiletin groups (Fig. 7a). The cluster analysis of heatmap manifested that genes correlated with autophagy, ferroptosis, and the NRF2 signaling pathway were significantly and differentially expressed between cisplatin and cisplatin-plus-nobiletin groups (Fig. 7b). The top 10 cellular processes of Kyoto Encyclopedia of Genes and Genomes (KEGG) enrichment analysis showed that DEGs were enriched in the autophagy, ferroptosis, and apoptosis pathways (Fig. 7c). The Gene Ontology (GO) annotation analysis showed that among the 10 most prominent GO terms of each GO category, the establishment of localization was the most significant biological process, and the DEGs were mainly annotated in vesicle of cellular components and metal ion binding of molecular functions, respectively (Fig. 7d). The top 30 pathways of KEGG enrichment analysis showed that the DEGs were enriched in the autophagy pathway (Fig. 7e). These findings indicated the pivotal impacts of autophagy and ferroptosis on the protective effect of nobiletin against cisplatin-induced ototoxicity.

## Discussion

Although cisplatin is the pioneer of anti-cancer drug, it leads to bilateral, cumulative, and permanent deafness, seriously affecting the quality of life^28^. Therefore, the new therapeutic agents that offer improved hearing protection and safety are required to prevent and ameliorate cisplatin-induced ototoxicity. In the present study, the results of CCK-8 assay exhibited that cisplatin diminished the survival rate of HEI-OC1 cells in dose-dependent manners, indicating that cisplatin could suppress the proliferation of HEI-OC1 cells. Previous studies have demonstrated that cisplatin triggered apoptosis of auditory cells, manifesting as karyopyknosis, DNA fragmentation, and caspase-3 activation^8,29^. In our study, the results of cleaved caspase-3 and TUNEL staining illustrated that cisplatin stimulus exacerbated the severity of cell loss, accompanied by evident nuclear condensation and intense apoptotic signals. These results suggested that the cytotoxicity of cisplatin was primarily triggered by apoptosis, instead of necrosis. Encouragingly, nobiletin considerably ameliorated cellular trauma caused by cisplatin and weakened the intensity of apoptotic signals, manifesting that nobiletin suppressed auditory cell apoptosis caused by cisplatin.

Increasing evidence has indicated that ROS overproduction is primarily responsible for the auditory cell impairment caused by cisplatin^2,30^. Excessive ROS could lead to the disruption or depletion of antioxidant defense system and damage the function of antioxidant enzymes, such as SOD, simultaneously creating lipid peroxides, such as 4-HNE^31^. Nobiletin was previously reported to mitigate oxidative stresses in response to diverse stimuli^32,33^. In our study, the results of DCFH-DA staining showed that nobiletin considerably decreased the production of intracellular ROS triggered by cisplatin. Furthermore, nobiletin effectively suppressed the elevation of 4-HNE in response to cisplatin and showed no influence on its expression in the absence of cisplatin. Previous researches indicated that cisplatin may directly inhibit the enzymatic activity of antioxidant gene products and/or stimulate the production of ROS, leading to a compensatory up-regulation of gene expression in response to oxidative stress^34,35^. In the present study, the treatment of cisplatin mildly elevated the level of SOD1, suggesting that cisplatin-induced oxidative stress may strengthen the antioxidative mechanisms. It is noteworthy that pretreatment with nobiletin significantly facilitated the up-regulation of SOD1, specifically in the cisplatin-plus-nobiletin group. Our discoveries revealed that nobiletin ameliorated oxidative stress caused by cisplatin in auditory cells.

It has been confirmed that LC3 serves as an autophagy indicator, while the transformation of soluble LC3-I into LC3-II is involved in the autophagosome synthesis, which represents the occurrence of autophagy^36^. Furthermore, the autophagy activation leads to the degradation of autophagic substrate SQSTM1/p62^37^. Moreover, cisplatin stimulus triggers autophagy, while further escalation of autophagic flux could resist auditory cell impairment^38,39^. In our study, the autophagy was up-regulated by the treatment of cisplatin, as evidenced by the elevated expression of LC3B-II and increased autophagosome synthesis and SQSTM1/p62 degradation. Nobiletin has been previously reported to activate autophagy by up-regulating the expression of LC3B in a concentration-dependent manner^40,41^. In the current study, the exclusive administration of nobiletin slightly increased the expression of LC3B-II compared to the control group. Remarkably, pretreatment with nobiletin before cisplatin exposure significantly enhanced the expression of LC3B-II and led to a substantial accumulation of spotted autophagosomes. Nevertheless, the pretreatment of nobiletin significantly elevated the level of SQSTM1/p62, which was characterized as an indication of autophagic degradation. It has been reported that the elevated level of SQSTM1/p62 obstructed KEAP1-NRF2 interplay, subsequently triggering nuclear translocation and transcription of NRF2 to facilitate its downstream signaling cascade^13^. Furthermore, NRF2 in turn activated the SQSTM1/p62 gene transcriptional regulation through a positive feedback loop^42^. Therefore, we hypothesized that the enhanced expression of SQSTM1/p62 was caused by the transcriptional activation of NRF2, which was verified by the augment of NRF2 and the expressions of its downstream genes after the pretreatment of nobiletin. These findings indicated that nobiletin alleviated cisplatin-induced auditory cell damage via activating autophagy.

Previous studies have demonstrated that NRF2 contributes to the regulation of antioxidant enzymes and plays cytoprotective roles to resist oxidative stresses^43^, while the transcriptional activation of NRF2 safeguarded auditory cells against cisplatin-elicited ototoxicity^44,45^. Numerous studies have shown that nobiletin activated NRF2 signaling pathway to resist oxidative stress triggered by hypoxia and nonalcoholic fatty liver diseases^32,33^. Furthermore, nobiletin has been demonstrated to directly augment the expression of NRF2 and HMOX1, even under physiological conditions in the absence of oxidative stress or mitochondrial dysfunction^46^. In the current study, there was a trivial increase in the expressions of nuclear NRF2 and HMOX1 following cisplatin exposure, possibly as compensatory reactions to combat oxidative stresses. In the absence of cisplatin, the treatment of nobiletin moderately enhanced the nuclear translocation of NRF2 and the expression of downstream antioxidant genes encoding *Hmox1* and *Nqo1*. Whereas in the cisplatin-plus-nobiletin group, the fluorescence signal of NRF2 was significantly intensified, accompanied by distinct nuclear translocation and synchronous elevation of both HMOX1 and NQO1. These findings indicated the potential of nobiletin to stimulate NRF2 signaling pathway in both HEI-OC1 cells and cochlear explants.

Ferroptosis is a distinct form of cell death characterized by morphological, biochemical, and genetic differences from apoptosis, autophagy, and necrosis. Z-VAD-FMK (an inhibitor of pan-caspase) and necrostatin-1 (a specific inhibitor of RIPK1) are ineffective in blocking ferroptosis^47^. In contrast, iron chelators such as deferoxamine and antioxidants such as Fer-1 can inhibit ferroptosis^48^. Previous studies have confirmed that ferroptosis is the upstream process of apoptosis in cisplatin-induced nephrotoxicity^49^. Our study demonstrated that Fer-1 could inhibit both cisplatin-induced ferroptosis and apoptosis, while Z-VAD-FMK could only suppress apoptosis but not ferroptosis, indicating that cisplatin-induced ferroptosis may precede and trigger apoptosis, and inhibition of ferroptosis attenuates apoptosis. Recent studies have confirmed that cisplatin-induced ferroptosis is an autophagy-dependent cell death process^50^. Autophagy triggers ferroptosis, known as ferritinophagy, by facilitating the binding of ferritin to lysosomes and inducing the degradation of ferritin^51^. Autophagy plays a crucial role in regulating the cellular iron homeostasis, and the autophagy receptor protein SQSTM1/p62 is involved in autophagy and the NRF2 pathway^52^. NRF2 has been demonstrated to inhibit ferroptosis through targeting genes related to glutathione synthesis/metabolism, such as *Gpx4* and *Slc7a11*, as well as genes involved in iron metabolism, such as *Fth* and *Ftl*^53^. The inactivation or degradation of GPX4 is regarded as a biomarker of ferroptosis. Depletion of GSH, a substrate of GPX4, can induce the accumulation of lipid peroxides, ultimately leading to ferroptotic cell death^54^. Ferroptosis is recognized as an essential mechanism of cisplatin-induced auditory cell impairment, while Fer-1 is revealed with protection of auditory cells against cisplatin stimulus^9^. Previous research has confirmed that nobiletin inhibited ferroptosis by activating NRF2/GPX4 signaling pathway^24^. In the present study, cisplatin diminished the levels of GPX4, SLC7A11, FTH, FTL, and GSH, whereas the addition of nobiletin significantly reversed this recession and ameliorated cisplatin-induced impairment, showing comparable effect to that of Fer-1. Meanwhile, nobiletin markedly accelerated the transcription of NRF2, along with its target genes encoding both *Hmox1* and *Nqo1*. Therefore, our study suggested that the inhibitory function of nobiletin on ferroptosis induced by cisplatin could be mediated through the NRF2/GPX4 pathway.

Finally, the results of RNA sequencing analysis revealed significant differences in gene expression correlated with autophagy, ferroptosis, and the NRF2 signaling pathway after the pretreatment of nobiletin compared with the treatment of cisplatin alone. Correspondingly, the KEGG pathway analysis revealed that DEGs were enriched in the autophagy and ferroptosis pathways. These findings further verified that the intrinsic mechanisms of the protection of nobiletin against cisplatin-induced ototoxicity are correlated with the levels of autophagy and ferroptosis.

In summary, our study demonstrated, for the first time, that nobiletin ameliorated cisplatin-induced ototoxicity via inhibiting apoptosis and attenuating oxidative stress. Mechanistically, the protective function of nobiletin is attributed to the activation of autophagy and the inhibition of NRF2/GPX4-mediated ferroptosis. This study implies that nobiletin could serve as a promising therapeutical option for preventing cisplatin-induced ototoxicity.

## Materials and methods

### Experimental animals

All experimental procedures complied with the ARRIVE guidelines (Animal Research: Reporting of In Vivo Experiments). All animal experiments were approved by the Laboratory Animal Management and Ethics Review Committee of Shandong Provincial Hospital and conducted per the requirements of the Shandong Provincial Hospital Animal Protection and Utilization Committee (Permit No. 2022-025). All methods were performed in accordance with relevant guidelines and regulations. C57BL/6 mice aged P4 were purchased from Spelford Biotechnology Co., Ltd. (Beijing, China). The animals were anesthetized and decapitated, followed by subsequent dissection and experiments.

### Cell culture

The HEI-OC1 cells were incubated in high-glucose DMEM (C11995500BT, Gibco, USA) containing 10% fetal bovine serum (16000044, Gibco, USA) at 33 ℃ and 10% CO2 of humidified atmosphere.

### Cochlea dissection and culture

The P4 C57BL/6 mice were sacrificed with their skulls opened to carefully extract their cochleae. The spiral ligament and stria vascularis were removed, and the basilar membrane was adhered to glass slides pre-coated with Cell-Tak (354240, BD Biosciences, USA) under a dissecting microscope. Then, the cochlear explants were cultured in DMEM/F12 medium (10565018, Thermo Fisher Scientific, USA) containing ampicillin (A1170, Solarbio, China), N-2 (17502048, Thermo Fisher Scientific, USA), and B-27 (17504044, Thermo Fisher Scientific, USA) under appropriate conditions (i.e., 37 °C and 5% CO2).

### Drug treatments

Cisplatin (P4394, Sigma-Aldrich, USA) was dissolved in saline, nobiletin (HY-N0155, MCE, USA), ferrostatin-1 (HY-100579, MCE, USA), and Z-VAD-FMK (HY-16658B, MCE, USA) were dissolved in DMSO. These solutions were diluted in culture medium. After incubation overnight, the cells and cochlear explants were treated with or without 20 µM nobiletin for 8 h, followed by exposure to 20 µM cisplatin for 24 h. Alternatively, cells and cochlear explants were treated with 20 µM nobiletin alone for 24 h. Furthermore, based on previous study^9^, 30 µM ferrostatin-1 and 40 µM Z-VAD-FMK were employed as inhibitors of ferroptosis and apoptosis, respectively, to pretreat cells for 2 h, followed by exposure to 20 µM cisplatin for 24 h.

### Cell viability assay

Cells were seeded in 96-well plates with 3 biological replicates and cultured overnight and treated with drugs. The CCK-8 solution (96992, Sigma-Aldrich, US) was supplemented to each well and incubated for 2 h. Then, the absorbance at 450 nm was detected via a microplate reader (SpectraMAX M2, CA, USA).

### Hair cell counting

After the cochlear hair cells were labelled with myosin 7a, the basal, middle, and apical regions of cochlea were imaged using a 40 × objective lens on a Leica TCS SP8 confocal fluorescence microscope (Leica Microsystems, Biberach, Germany) and the immunostaining positive cells were quantified using ImageJ software. The number of hair cells per 250 µm were calculated in three cochlear segments, i.e., apical (apex), middle (mid), and basal (base).

### Detection of reactive oxygen species

The intracellular ROS was detected via DCFH-DA staining (S0033S, Beyotime Biotechnology, China). Cells or tissues under different treatments were stained by 10 µM DCFH-DA for 30 min in an incubator at 37 °C. Nuclei were stained with Hoechst 33258 (C0021, Solarbio, China) or DAPI (D9542, Sigma-Aldrich, USA) for 10 min in an incubator at 37 °C. Fluorescence images were captured using the confocal microscope.

### Detection of glutathione level

The intracellular GSH levels were measured using the Glutathione Assay Kit (S0052, Beyotime Biotechnology, China). Following various treatments, cells were harvested by centrifugation. Then, cell lysates were prepared and incubated with the assay solution for 25 min at room temperature. The absorbance at 412 nm was detected via a microplate reader (SpectraMAX M2, CA, USA).

### Immunofluorescence

Cells or tissues were fixed for 30 min with 4% paraformaldehyde (PFA) and permeabilized for 30 min with either 0.2% or 1% Triton X-100, respectively, followed by blocking for 1 h in PBS mixed with 0.1% Triton X-100, 5% donkey serum, and 1% bovine serum albumin (BSA). Then, the samples were incubated with the anti-Myosin 7a antibody (138-1, DSHB, USA), anti-cleaved caspase 3 antibody (9664s, CST, USA), anti-LC3B antibody (3868s, CST, USA), anti-NRF2 antibody (12721s, CST, USA), and anti-GPX4 antibody (ab125066, Abcam, USA) at a 1:500 dilution overnight at 4 °C. Then, the specimens were washed 3 times using PBS and incubated for 1 h with secondary fluorescent antibodies. All specimens were visualized with the images taken using the confocal microscope. The fluorescence intensity was measured by ImageJ software.

### TUNEL assay

The apoptosis of cells and tissues was detected using TUNEL Assay Kit (MA0224, Meilunbio, China) by following the manufacturer’s protocol, which was accompanied by additional staining of DAPI and any required antibodies. The samples were visualized with the images taken using the confocal microscope.

### Western blotting

HEI-OC1 cells were lysed by RIPA lysis buffer (P0013, Beyotime Biotechnology, China) with protease inhibitor (ST506, Beyotime Biotechnology, China). After centrifugation, the nuclear and cytoplasmic proteins were extracted using the Nuclear and Cytoplasmic Protein Extraction Kit (P0027, Beyotime Biotechnology, China). Then, the protein concentration was measured using BCA Protein Assay Kit (PC0020, Solarbio, China). Following denaturation, proteins were fractionated via 12% SDS-PAGE and subsequently transferred to polyvinylidene fluoride membranes (ISEQ00010, Merck Millipore, China). After blocking with 5% skimmed milk in TBS containing 0.05% tween20 (TBST) for 1 h at room temperature, the blots were cut prior to hybridization with antibodies. Subsequently, the membranes were probed with primary antibodies overnight at 4 °C, followed by incubation with secondary antibodies for 1 h at room temperature. Finally, the immunoblots were performed with the enhanced chemiluminescence system (Tanon5200, China) and quantified with ImageJ software. The primary antibodies included anti-cleaved caspase 3 antibody (9664s, CST, USA), anti-SOD1 antibody (A12537, ABclonal, China), anti-4-HNE antibody (ab46545, Abcam, USA), anti-LC3B antibody (3868s, CST, USA), anti-SQSTM1/p62 antibody (ab109012, Abcam, USA), anti-NRF2 antibody (A1244, ABclonal, China), anti-HMOX1 antibody (A1346, ABclonal, China), anti-NQO1 antibody (A19586, ABclonal, China), anti-GPX4 antibody (ab125066, Abcam, USA), anti-SLC7A11 antibody (ab175186, Abcam, USA), anti-FTH antibody (ab183781, Abcam, USA), anti-FTL antibody (ab109373, Abcam, USA), anti-Histone H3 antibody (ab1791, Abcam, USA), and anti-β-actin (Ta-09, ZSGB-BIO, China), all were used at a 1:1000 dilution.

### Quantitative real-time PCR

Total RNA was extracted from HEI-OC1 cells after different treatments using RNAiso Plus (9108, Takara, China) and reverse transcribed to cDNA by Evo M-MLV RT Mix Kit (AG11728, Accurate Biology, China). The qPCR was performed using SYBR Green Premix Pro Taq HS qPCR Kit (AG11701, Accurate Biology, China) on a QuantStudio5 Real-Time PCR system (Thermo Fisher Scientific, USA). The primers and their sequences using in the qPCR were provided in Table 1.

**Table 1:** Primers and their sequences used for qPCR.

Primer	Primer sequence
*Gapdh* forward	5′-AAATGGTGAAGGTCGGTGTGAAC-3′
*Gapdh* reverse	5′-CAACAATCTCCACTTTGCCACTG-3′
*Sod1* forward	5′-GGAACCATCCACTTCGAGCAG-3′
*Sod1* reverse	5′-ACAGCCTTGTGTATTGTCCCC-3′
*Lc3b* forward	5′-CATGCCGTCCGAGAAGACCT-3′
*Lc3b* reverse	5′-GATGAGCCGGACATCTTCCACT-3′
*Sqstm1/p62* forward	5′-CCTCAGCCCTCTAGGCATTG-3′
*Sqstm1/p62* reverse	5′-TTCTGGGGTAGTGGGTGTCA-3′
*Nrf2* forward	5′-TAGATGACCATGAGTCGCTTGC-3′
*Nrf2* reverse	5′-GCCAAACTTGCTCCATGTCC-3′
*Hmox1* forward	5′-CTGGAGATGACACCTGAGGTCAA-3′
*Hmox1* reverse	5′-CTGACGAAGTGACGCCATCTG-3′
*Nqo1* forward	5′-TGGCCGAACACAAGAAGCTG-3′
*Nqo1* reverse	5′-GCTACGAGCACTCTCTCAAACC-3′

### RNA sequencing analysis

Total RNA was extracted from HEI-OC1 cells using RNAiso Plus (9108, Takara, China). The cDNA libraries were established by Hieff NGS Ultima Dual-mode mRNA Library Prep Kit for Illumina (Yeasen, China) and sequenced on Illumina NovaSeq 6000 (Illumina, USA). The clustering of each sample was performed using the Pearson correlation and principal component analysis (PCA). DEGs were identified and analyzed by DESeq2 software, with the significant difference determined by *p*-value < 0.05 and fold change ≥ 2. The volcanic map and heatmap were generated in Hiplot Pro (https://hiplot.com.cn/). Both GO annotation and KEGG enrichment analysis^55,56^ were conducted in Sangerbox platform (http://vip.sangerbox.com/).

### Statistical analyses

Data were presented as mean ± standard deviation (SD) based on at least three biological replicates. All statistics were analyzed using one-way ANOVA with Dunnett’s multiple comparisons test and two-sided unpaired t-test performed by GraphPad Prism 8 software. The statistically significance was determined based on *P* < 0.05.

### Ethical approval

All animal experiments were approved by the Laboratory Animal Management and Ethics Review Committee of Shandong Provincial Hospital (Permit No. 2022-025). All methods were performed in accordance with relevant guidelines and regulations. All experimental procedures complied with the ARRIVE guidelines.

### Supplementary Information

Supplementary Information.

## Data Availability

The data presented in this study are included in the article/Supplementary Material. The datasets generated or analysed during the current study are available in the National Center for Biotechnology Information repository, https://www.ncbi.nlm.nih.gov/sra/, with the accession number PRJNA1022596.
