# Supplementary material for: Nobiletin alleviates cisplatin-induced ototoxicity via activating autophagy and inhibiting NRF2/GPX4-mediated ferroptosis
Source: Sci Rep. 2024 Apr 3;14:7889. doi: 10.1038/s41598-024-55614-4 (PMC10991266; doi:10.1038/s41598-024-55614-4)
Supplement: Supplementary file 1 — Supplementary Information. [file 41598_2024_55614_MOESM1_ESM.docx]

Nobiletin alleviates cisplatin-induced ototoxicity via activating autophagy and inhibiting NRF2/GPX4-mediated ferroptosis

# Wenao Song1, Li Zhang2, Xiaolin Cui1, Rongrong Wang1, Jingyu Ma2, Yue Xu4, Yan Jin2, Dawei Wang3,*, and Zhiming Lu1,2,*

1Department of Clinical Laboratory, Shandong Provincial Hospital, Shandong University, Jinan, 250021, China

2Department of Clinical Laboratory, Shandong Provincial Hospital Affiliated to Shandong First Medical University, Jinan, 250021, China

3Department of Orthopedic, Shandong Provincial Hospital Affiliated to Shandong First Medical University, Jinan, 250021, China

4Department of Otolaryngology-Head and Neck Surgery, Shandong Provincial Hospital, Shandong University, Jinan, 250021, China

*[corresponding. Zhiming Lu: luzhiming@sdu.edu.cn](mailto:corresponding.author@email.example); Dawei Wang: wangdawei@sdu.edu.cn

#
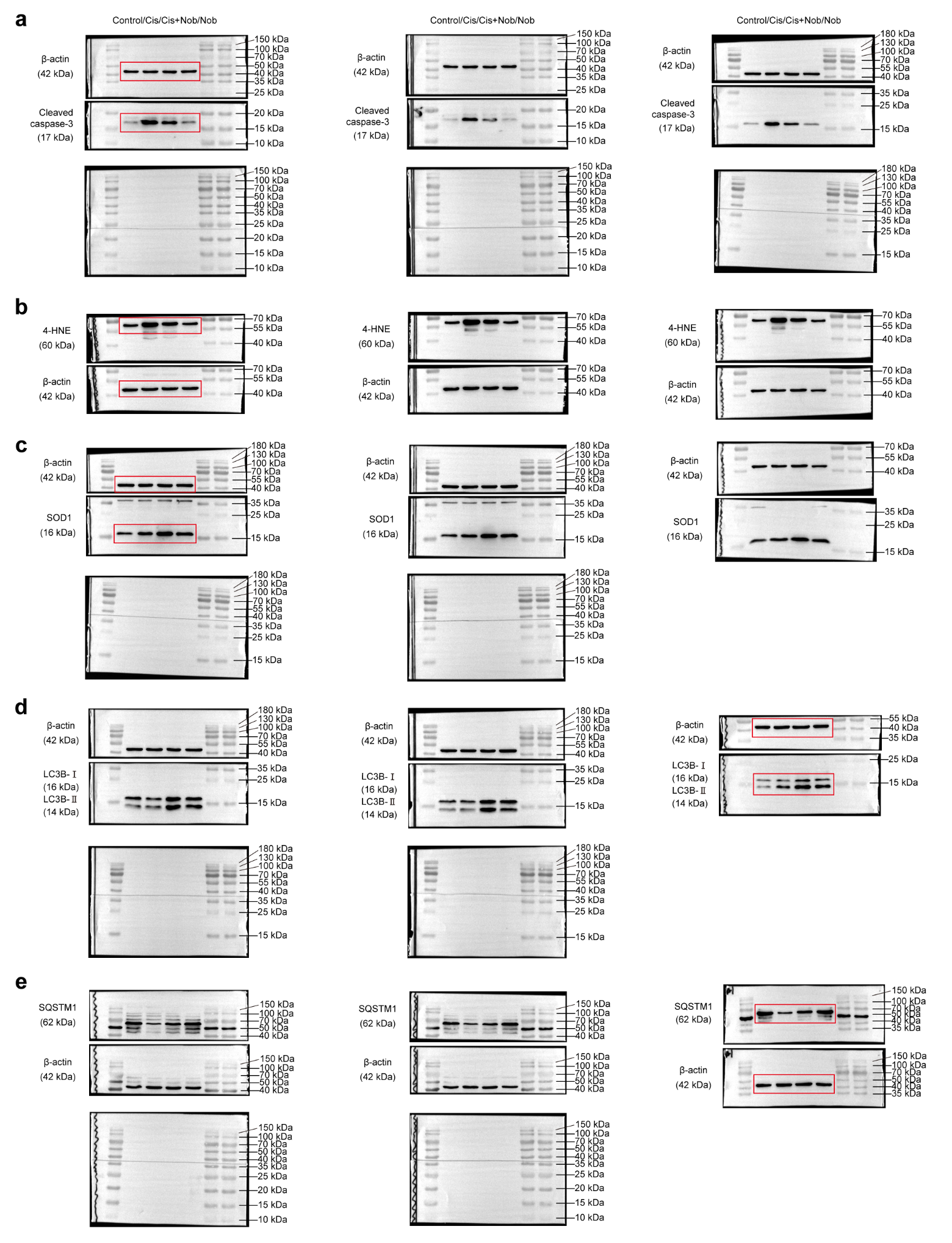


**Figure S1.** The original western blot images. The original blot area used in the main image is indicated by a red box. (a) The Western blot images corresponding to Figure 2a. (b) The Western blot images corresponding to Figure 3e. (c) The Western blot images corresponding to Figure 3g. (d) The Western blot images corresponding to Figure 4a. (e) The Western blot images corresponding to Figure 4d.


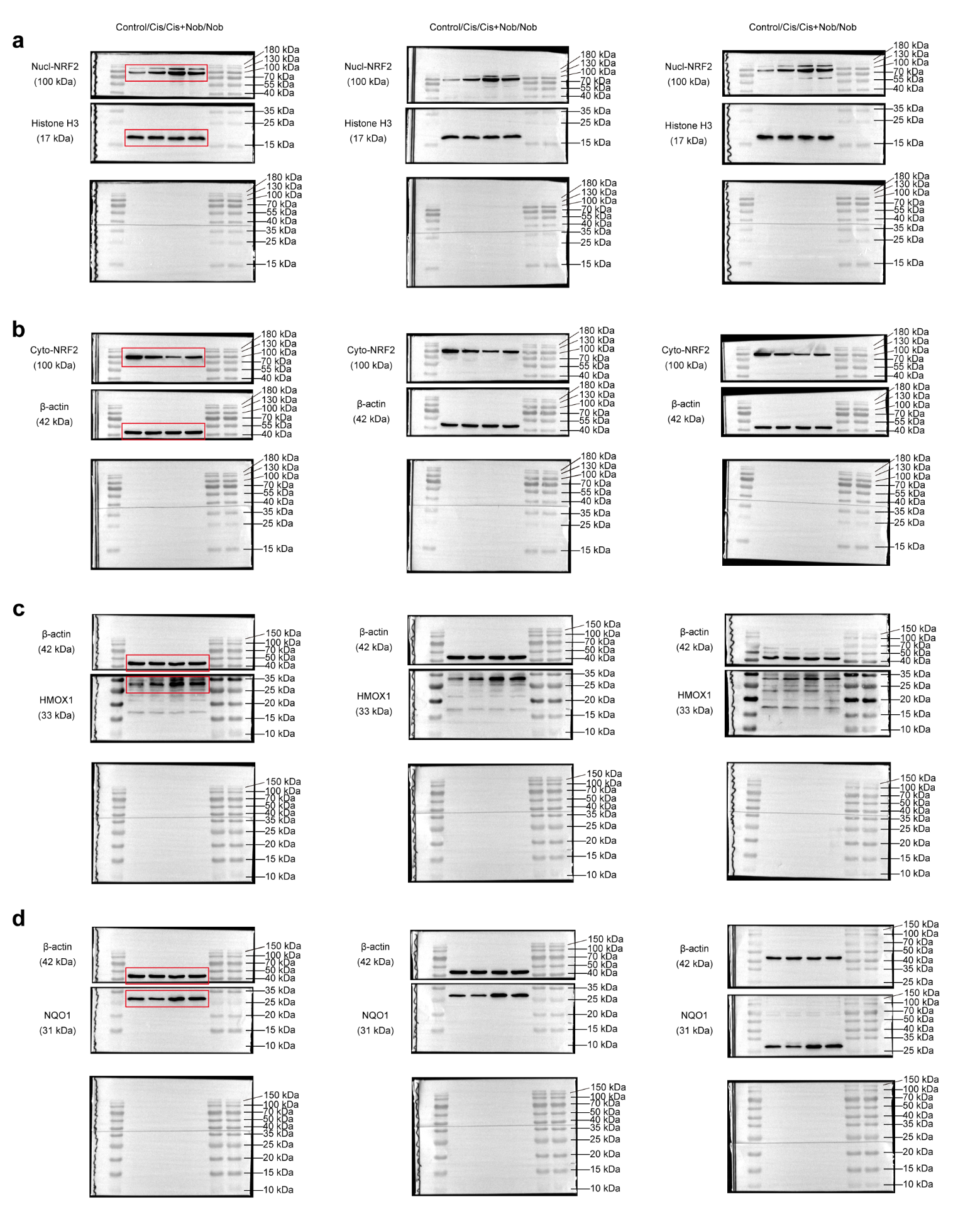


**Figure S2.** The original western blot images. The original blot area used in the main image is indicated by a red box. (a) The Western blot images corresponding to Figure 5a. (b) The Western blot images corresponding to Figure 5a. (c) The Western blot images corresponding to Figure 5d. (d) The Western blot images corresponding to Figure 5d.


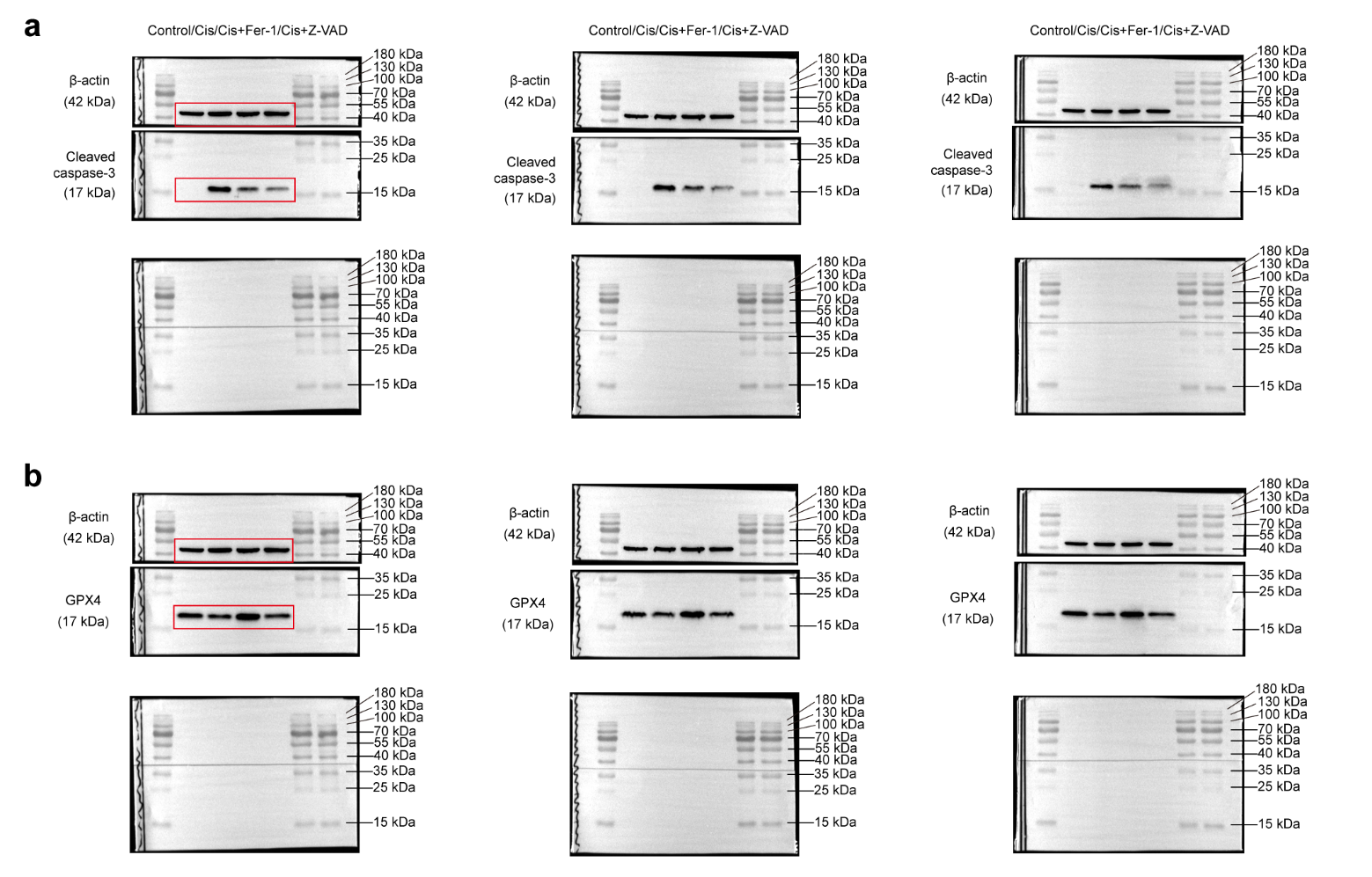


**Figure S3.** The original western blot images. The original blot area used in the main image is indicated by a red box. (a) The Western blot images corresponding to Figure 6a. (b) The Western blot images corresponding to Figure 6a.


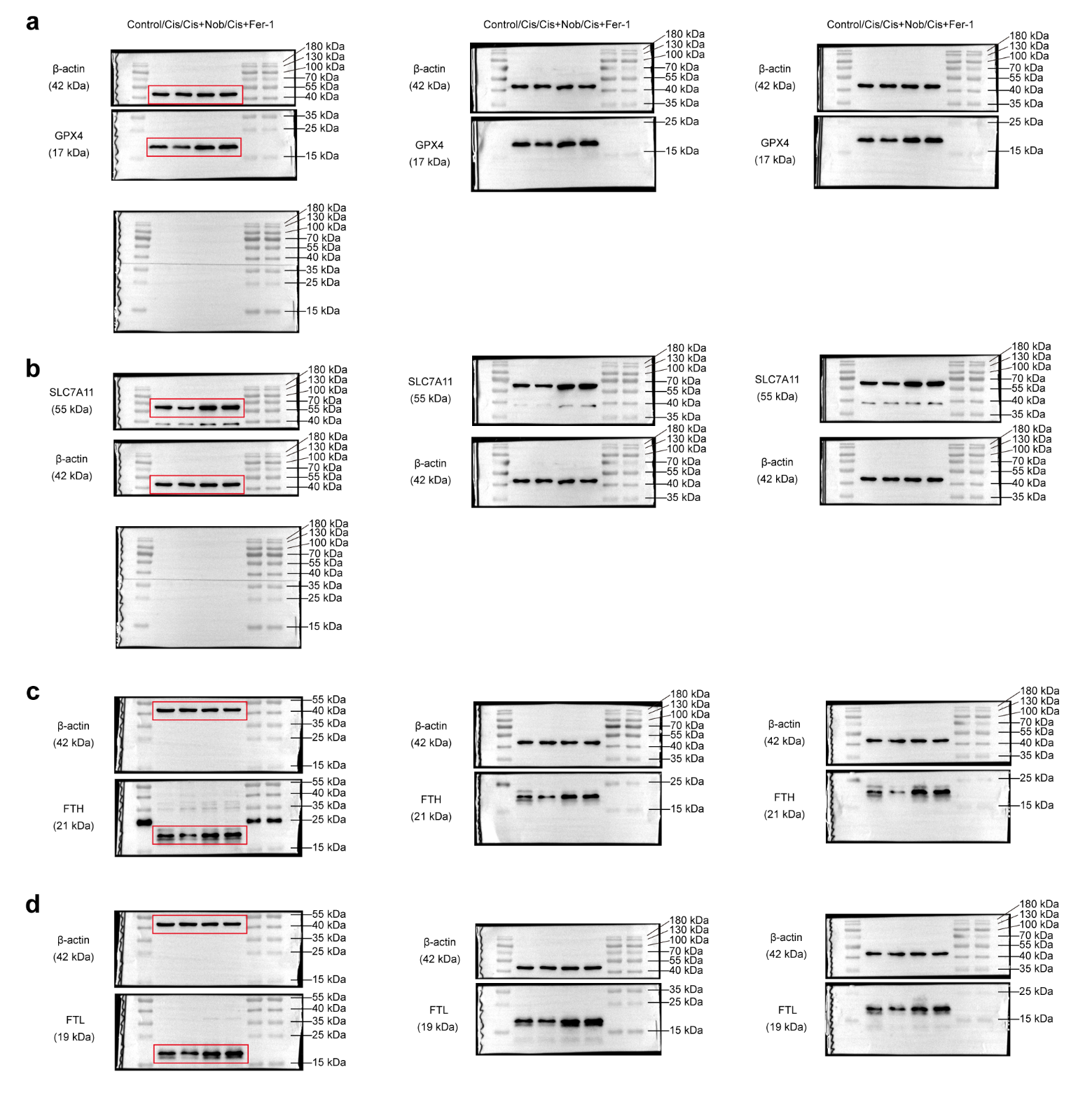


**Figure S4.** The original western blot images. The original blot area used in the main image is indicated by a red box. (a) The Western blot images corresponding to Figure 6d. (b) The Western blot images corresponding to Figure 6d. (c) The Western blot images corresponding to Figure 6g. (d) The Western blot images corresponding to Figure 6g.
